# Supplementary material for: MAVS signaling is required for preventing persistent chikungunya heart infection and chronic vascular tissue inflammation
Source: Nat Commun. 2023 Aug 3;14:4668. doi: 10.1038/s41467-023-40047-w (PMC10400619; doi:10.1038/s41467-023-40047-w)
Supplement: Supplementary file 1 — Supplementary Information [file 41467_2023_40047_MOESM1_ESM.pdf]

MAVS signaling is required for preventing persistent chikungunya heart infection and chronic vascular tissue inflammation

Maria G. Noval[1,7], Sophie N. Spector[1], Eric Bartnicki[1], Franco Izzo[2,3], Navneet Narula[4], Stephen T. Yeung[1], Payal Damani-Yokota[1], M. Zahidunnabi Dewan[4], Valeria Mezzano[4], Bruno A. Rodriguez-Rodriguez[1], Cynthia Loomis[4,5], Kamal M. Khanna[1,6], and Kenneth A. Stapleford[1,7]

[1]Department of Microbiology, New York University Grossman School of Medicine, New York, NY, USA
[2]New York Genome Center, New York, NY, USA
[3]Division of Hematology and Medical Oncology, Department of Medicine and Meyer Cancer Center, Weill Cornell Medicine, New York, NY, USA
[4]Department of Pathology, New York University Grossman School of Medicine, New York, NY, USA
[5]Division of Advanced Research Technologies, New York University Grossman School of Medicine, New York, NY, USA
[6]Perlmutter Cancer Center, New York University Grossman School of Medicine, New York, NY, USA

[7]These authors jointly supervised this work: Maria G. Noval. maria.noval@nyulangone.org and Kenneth A. Stapleford. kenneth.stapleford@nyulangone.org

Supplementary Fig.1. (Related to Fig. 1)

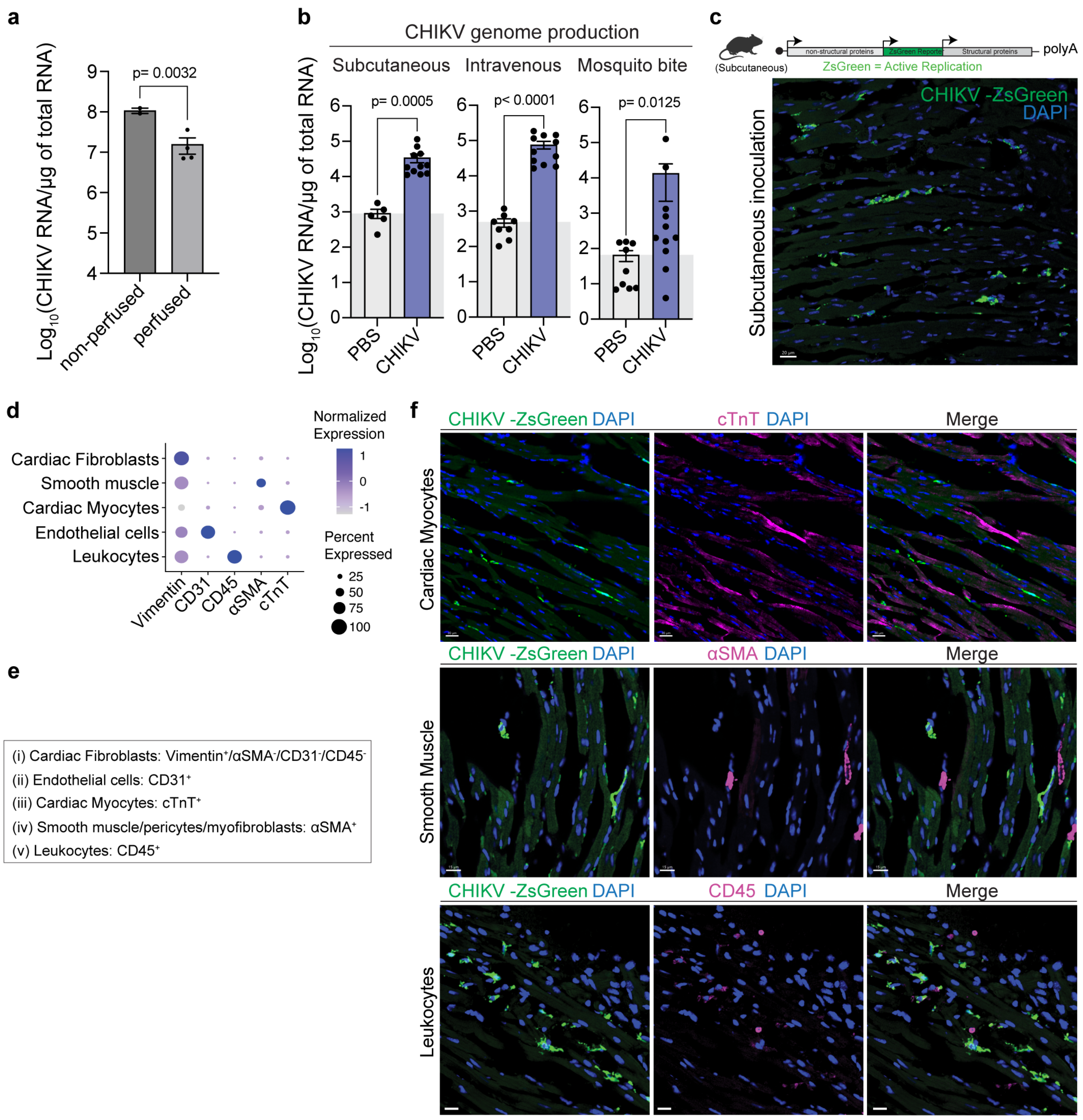

Supplementary Fig. 1, related to Fig. 1. a. *Ifnar1*$^{-/-}$ mice were infected intravenously with 1E2 PFU of CHIKV and harvested at 2 dpi. Animals were either perfused with more than 20 mL of PBS (perfused) or left untreated (non-perfused). Viral RNA was extracted from cardiac tissue homogenates and CHIKV genomes were quantified by RT-qPCR. Non-perfused (n=2), and perfused (n=4). Data from 1 independent experiment is represented as mean +/- SEM. P value was determined two-tailed unpair t-test. **b.** Six-week-old C57BL/6 mice were inoculated with 1E5 PFU of CHIKV or mock-infected (PBS) subcutaneously, intravenously, or via natural transmission through a mosquito bite. Mice were harvested at 2 dpi for subcutaneous and intravenous infection routes, and at 2 and 3 dpi for mice infected through mosquito bite. Animals were perfused with 30 ml of cold PBS. Viral RNA genomes in heart tissue were quantified using RT-qPCR. Data from 3 independent experiments are represented as mean +/- SEM. For subcutaneous inoculation: CHIKV-infected (n=11) and mock-infected (n=5). Intravenous inoculation: CHIKV-infected (n=11) and mock-infected (n=8). Mosquito bite: CHIKV-infected (n=11) and mock-infected (n=9). p values were calculated using two-tailed Mann Whitney test. The gray box represents the background RNA levels determined for mock-infected controls. **c.** Mice were inoculated subcutaneously with 1E5 PFU of reporter CHIKV-ZsGreen or PBS-control. Scale= 20 µm. Blue channel: DAPI. Green channel: CHIKV-infected cells. **d.** Dot plot showing the expression levels of the markers used in the study by cardiac cell type using the *Tabula Muris* data set. **e.** Strategy for cell-type identification. **f.** Representative fluorescence microscopy images from 3 independent experiments of ventricular sections of CHIKVinfected hearts stained with different cardiac cell type markers. Cardiac myocytes (Scale= 20 µm), smooth muscle and leukocytes (Scale= 15 µm). Created with BioRender.com. Source data are provided as a source data file.

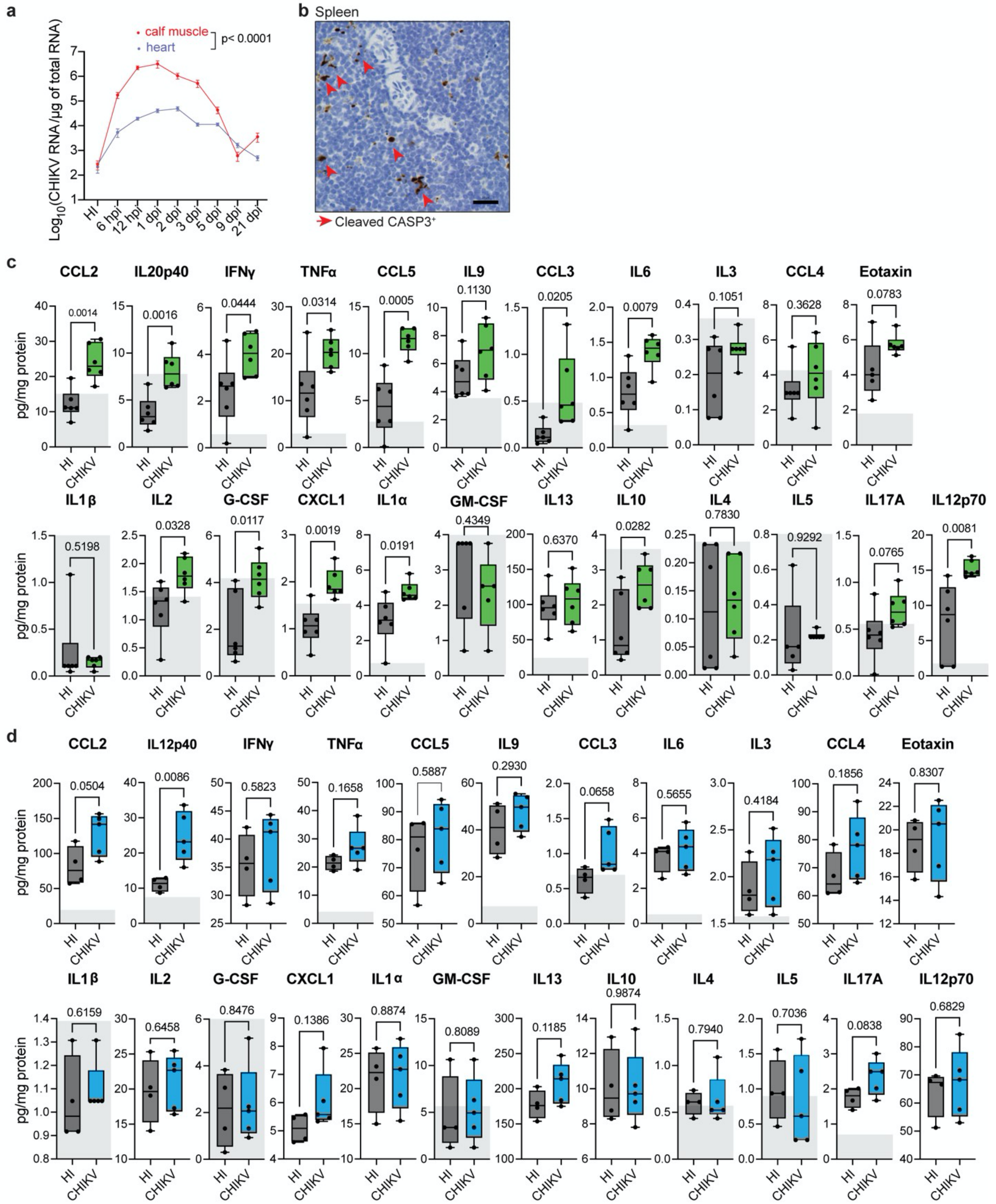

Supplementary Fig. 2, related to Fig. 2. a. CHIKV replication kinetics in the heart (blue) and the calf muscle (red) measured by RT-qPCR. Each time point represents the mean +/- SEM. The gray box represents the background RNA levels determined for mock-infected controls (PBS). For heart tissue homogenates: HI-control (n=39), 6 hpi (n=5), 12 hpi (n=5), 1 dpi (n=9), 2 dpi (n=18), 3 dpi (n=7), 5 dpi (n=13), 9 dpi (n=8) and 21 dpi (n=8). For calf muscle homogenates: HI-control (n=21), 6 hpi (n=5), 12 hpi (n=5), 1 dpi (n=9), 2 dpi (n=8), 3 dpi (n=7), 5 dpi (n=4), 9 dpi (n=4), and 21 dpi (n=8). **b**. Positive control signal of cleaved CASP3 antibody staining used for IHC (representative image from at least three repetitions). Scale= 20 µm. **c-d.** Individual values from which **Fig.2d** was generated. Each bar graph represents the protein levels in cardiac tissue homogenates for different analytes from the Bio-Plex Pro Mouse Cytokine 23-plex Assay. Values within the gray shading represent concentrations of the analyte that were beyond the calibration curve and therefore determined by extrapolation of the curve by the Luminex Software. Mock: HI inoculum. 2 dpi: HI-control and CHIKV-infected hearts (n=6/group). 5 dpi: HIcontrol (n=4) and CHIKV-infected hearts (n=5). Boxplots show median and quartile ranges, whiskers represent the range**.** P values were calculated using mixed-effects model test **(a)** or using two-tailed Mann-Whitney test **(c-d)**. Source data are provided as a source data file.

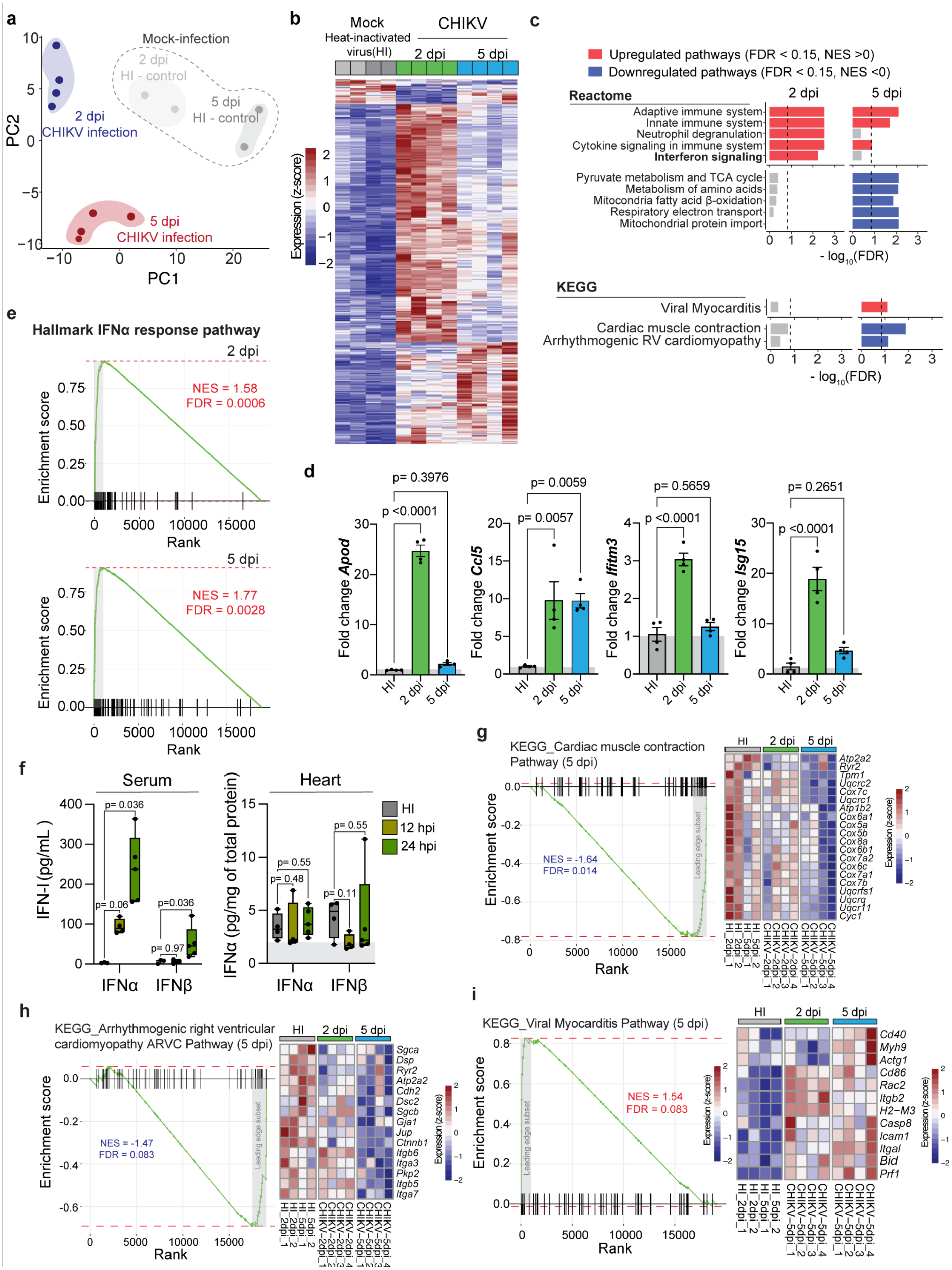

a
Mock-infection
2 dpi
HI - control
5 dpi
HI - control
2 dpi
CHIKV infection
5 dpi
CHIKV infection
PC1
PC2
b
Mock
Heat-inactivated
virus(HI)
CHIKV
2 dpi
5 dpi
Expression (z-score)
c
Upregulated pathways (FDR < 0.15, NES >0)
Downregulated pathways (FDR < 0.15, NES <0)
Reactome
2 dpi
5 dpi
Adaptive immune system
Innate immune system
Neutrophil degranulation
Cytokine signaling in immune system
Interferon signaling
Pyruvate metabolism and TCA cycle
Metabolism of amino acids
Mitochondria fatty acid β-oxidation
Respiratory electron transport
Mitochondrial protein import
- log10(FDR)
KEGG
Viral Myocarditis
Cardiac muscle contraction
Arrhythmogenic RV cardiomyopathy
- log10(FDR)
d
p= 0.3976
p <0.0001
Fold change Apod
p= 0.0059
p= 0.0057
Fold change Ccl5
p= 0.5659
p <0.0001
Fold change Ifitm3
p= 0.2651
p <0.0001
Fold change Isg15
HI
2 dpi
5 dpi
e
Hallmark IFNα response pathway
2 dpi
NES = 1.58
FDR = 0.0006
5 dpi
NES = 1.77
FDR = 0.0028
Enrichment score
Rank
f
Serum
Heart
IFN-I (pg/mL)
IFNα (pg/mg of total protein)
p= 0.036
p= 0.06
p=0.036
p= 0.97
p= 0.55
p= 0.48
p= 0.55
p= 0.11
IFNα
IFNβ
HI
12 hpi
24 hpi
g
KEGG_Cardiac muscle contraction
Pathway (5 dpi)
NES = -1.64
FDR= 0.014
Leading edge subset
Enrichment score
Rank
Atp2a2
Ryr2
Tpm1
Uqcrc2
Cox7c
Uqcrc1
Atp1b2
Cox6a1
Cox5a
Cox5b
Cox8a
Cox6b1
Cox7a2
Cox6c
Cox7a1
Cox7b
Uqcrfs1
Uqcrq
Uqcr11
Cyc1
Expression (z-score)
h
KEGG_Arrhythmogenic right ventricular
cardiomyopathy ARVC Pathway (5 dpi)
NES = -1.47
FDR = 0.083
Sgca
Dsp
Ryr2
Atp2a2
Cdh2
Dsc2
Sgcb
Gja1
Jup
Ctnnb1
Itgb6
Itga3
Pkp2
Itgb5
Itga7
i
KEGG_Viral Myocarditis Pathway (5 dpi)
NES = 1.54
FDR = 0.083
Cd40
Myh9
Actg1
Cd86
Rac2
Itgb2
H2-M3
Casp8
Icam1
Itgal
Bid
Prf1
HI_2dpi_1
HI_2dpi_2
HI_5dpi_1
HI_5dpi_2
CHIKV-2dpi_1
CHIKV-2dpi_2
CHIKV-2dpi_3
CHIKV-2dpi_4
CHIKV-5dpi_1
CHIKV-5dpi_2
CHIKV-5dpi_3
CHIKV-5dpi_4

Supplementary Fig. 3, related to Fig. 2 and Fig.3. a. Principal component analysis (PCA) showing that biological replicates clustered by treatment. **b.** Heatmap showing differentially expressed genes (FDR < 0.15 and absolute log_2 FC > 1). The boxes on top indicate the biological replicates for mock (heat-inactivated virus; n=4; grey boxes) and CHIKV-infected at either 2 dpi (n=4; green boxes) or 5 dpi (n=4; blue boxes). **c.** GSEA pathway enrichment analysis for Reactome, and KEGG datasets showing top and downregulated pathways at 2 dpi and 5 dpi. **d.** Validation of RNAseq targets. *Apod, Ccl5, Ifitim3,* and *Isg15* expression levels from CHIKV-infected and mock-infected (HI-inoculum) heart homogenates were measured by RT-qPCR at 2 and 5 dpi. Gray boxes indicate fold change of 1. HI-control (n=4), 2dpi (n=4) and 5 dpi (n=4). Data represent mean +/- SEM. **e**. Enrichment plot for the IFNα response pathway (Hallmark) at 2 dpi (upper panel) and 5 dpi (bottom panel). **f**. Protein levels of IFN-α and INF-β in serum (left panel) and heart tissue homogenates (right panel) at 2 dpi. Values within the gray shading represent concentrations of the analyte that were beyond the calibration curve and therefore determined by extrapolation of the curve by the Luminex Software. For serum: HI-control (n=3), 12 hpi (n=4) and 24 hpi (n=5). For heart tissue homogenates: HI-control (n=4), 12 hpi (n=4) and 24 hpi (n=5). Boxplots show median and quartile ranges, whiskers represent the range**.** Dataset at 24 hpi are the same as the dataset for WT at 24 hpi in Fig.4h-i. **(g-i)** Enrichment plot for the cardiac muscle contraction (KEGG) **(g)**, viral arrhythmogenic right ventricular cardiomyopathy (KEGG) at 5 dpi **(h)**, and the viral myocarditis pathway (KEGG) **(i)** at 5 dpi (left panel) and heatmaps indicating expression of the leading-edge genes in mock or CHIKV-infected mice at 2 dpi or 5 dpi. n=4 mice/group. **(e-h)**. NES = normalized enrichment score; grey shading indicates the genes belonging to the leading edge. P values were calculated one-way ANOVA with Dunnett's multiple comparison test **(d)** and using two-tailed Mann-Whitney test **(f)**.

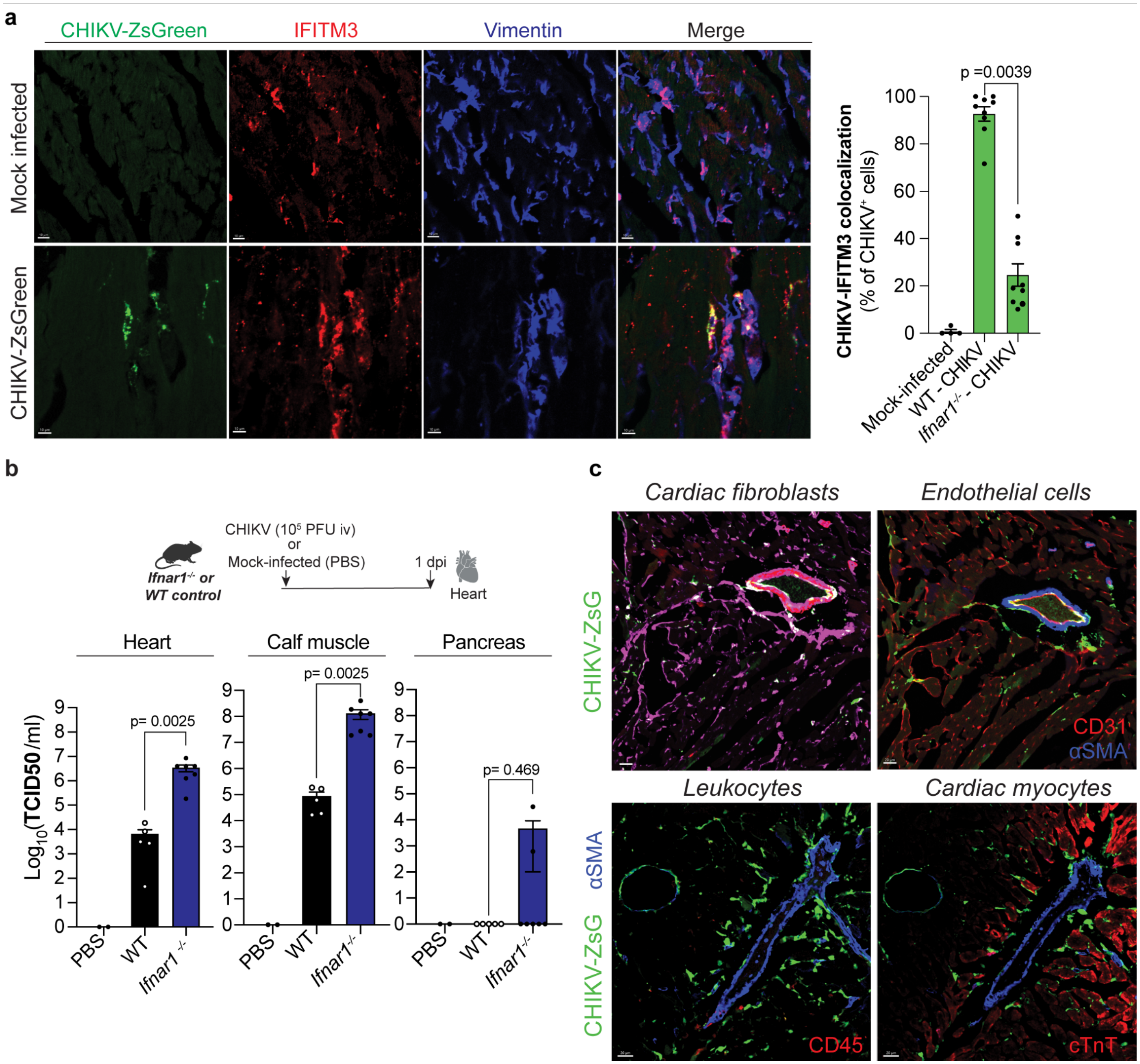

Supplementary Fig. 4, related to Fig.3. a. WT and *Ifnar1*$^{-/-}$ mice were inoculated intravenously with 1E5 PFU of reporter CHIKV-ZsGreen or mock-infected with PBS, harvested at 2 dpi, and cardiac tissue was stained with antiIFITM3 and anti-vimentin antibodies. Left panel: Representative fluorescence microscopy images from two independent repetitions showing ventricular sections of CHIKV-infected hearts and PBS control. Scale = 10 μm. Right panel: Colocalization analysis between CHIKV-infected cells and IFITM3 marker for WT and *Ifnar1*$^{-/-}$ infected or mock-infected mice. PBS control: 2 independent fields for n=2 mice. CHIKV-infected WT: 4-5 independent fields for n=2 mice. CHIKVinfected *Ifnar1*$^{-/-}$ mice: 4-5 independent fields for n=2 mice. Data are represented as the percentage of the total infected cells overlapping with the IFITM3 maker. **B.** Isolation of CHIKV infectious particles from heart, calf muscle and pancreas of WT or *Ifnar1*$^{-/-}$ mice. Mice were infected intravenously with 1E5 PFU of CHIKV or PBS. CHIKV infectious particles were determined by $TCID_{50}$ in BHK-21 cells. PBS-control (n=2), CHIKV-infected WT (n=5), and CHIKV-infected *Ifnar1*$^{/-}$ mice (n=7). **c.** Representative fluorescence microscopy images from two independent repetitions showing ventricular sections of *Ifnar1*$^{-/-}$ infected hearts colocalizing with markers of cardiac fibroblast, endothelial cells, leukocytes, and cardiac myocytes. Scale= 20 μm. **d.** Data are represented as mean +/- SEM (**a-b**). P values were calculated using twotailed Wilcoxon test **(a)** and two-tailed Mann-Whitney test **(b)**. Created with BioRender.com. Source data are provided as a source data file.

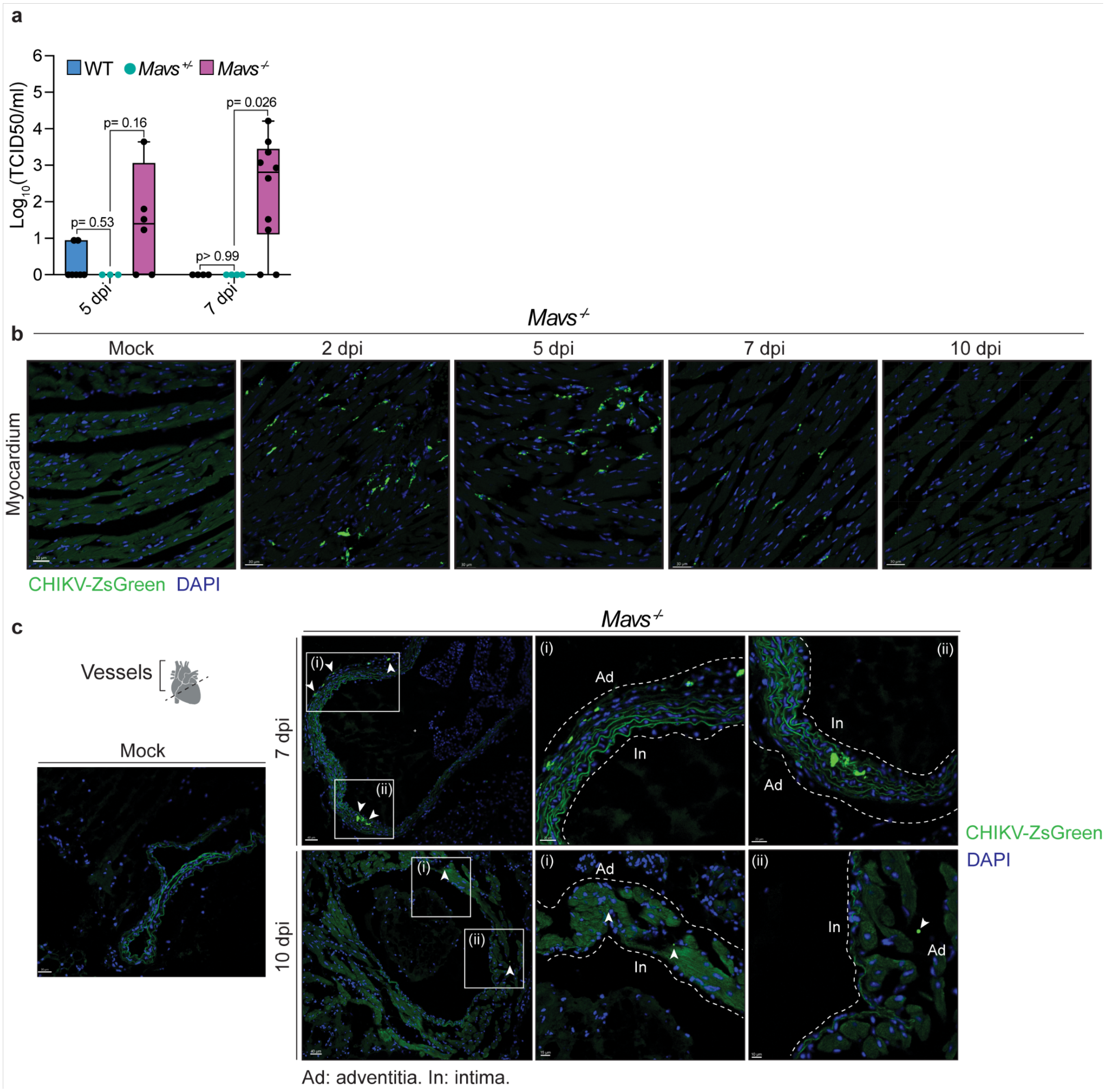

Supplementary Fig. 5, related to Fig.4. a. CHIKV cardiac infection kinetics in *Mavs*$^{-/-}$, *Mavs*$^{+/-}$and WT mice. Mice were infected intravenously with 1E5 PFU of CHIKV. CHIKV infectious particles from heart homogenates were determined by $TCID_{50}$ in BHK-21. For 5 dpi: WT (n=7), *Mavs*$^{+/-}$ (n=3), and *Mavs*$^{-/-}$ (n=6). For 7 dpi: WT (n=4), *Mavs*$^{+/-}$ (n=4), and *Mavs*$^{-/-}$ (n=10). Boxplots show median and quartile ranges, whiskers represent the range**.** Data set from *Mavs*$^{-/-}$ and WT mice are the same from **Fig 4b** at time point 5 and 7 dpi. **b.** Representative fluorescent microscopy images from two independent repetitions showing ventricular sections of CHIKV-ZsGreen infected *Mavs*$^{-/-}$ hearts. Scale=30 µm. **c.** Representative fluorescent microscopy images from two independent repetitions showing vessels of CHIKV-ZsGreen infected *Mavs*$^{-/-}$ hearts. Scale= 10-40 µm. P values were calculated using two-tailed multiple Mann-Whitney tests **(a)**. Created with BioRender.com. Source data are provided as a source data file.

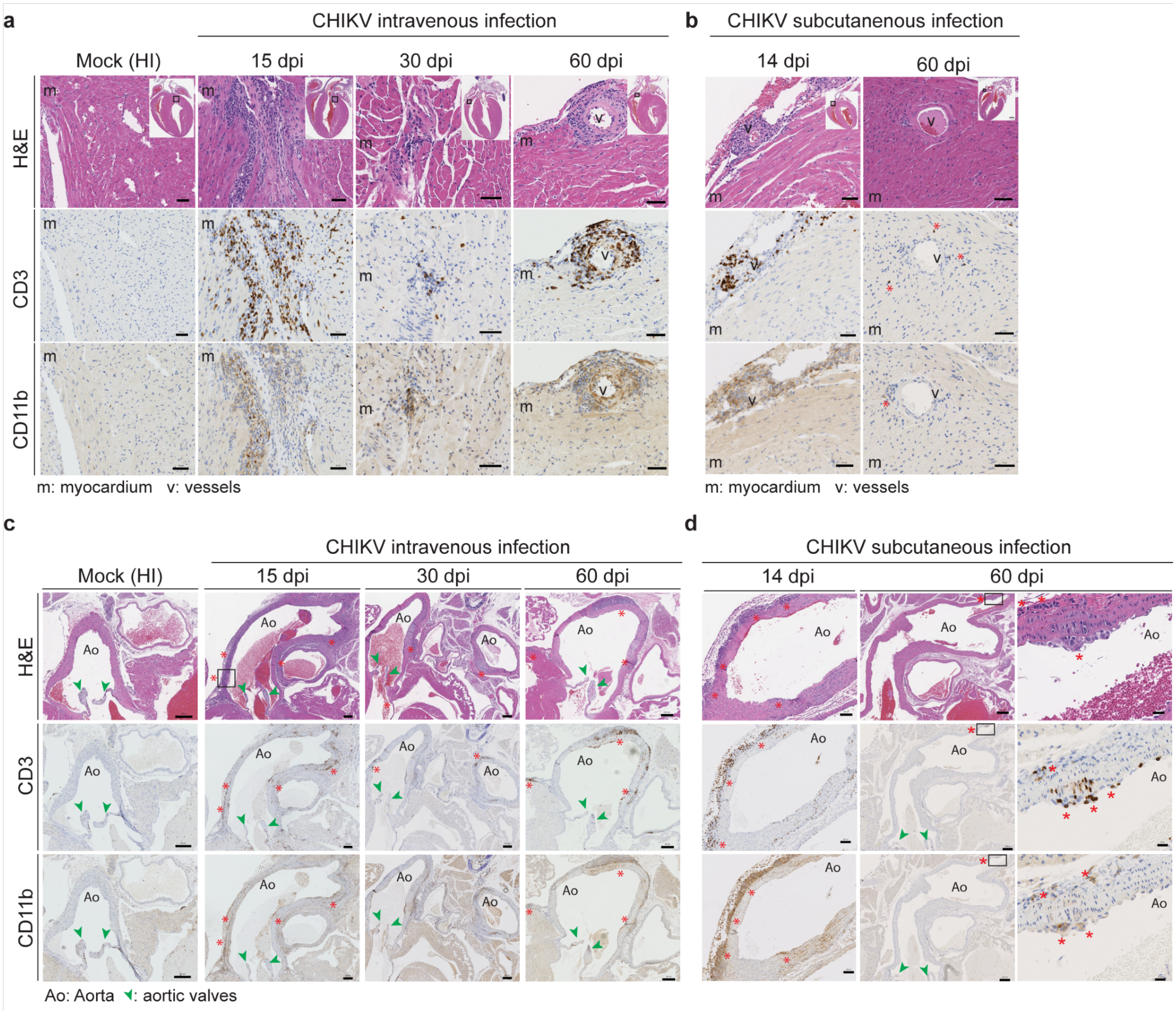

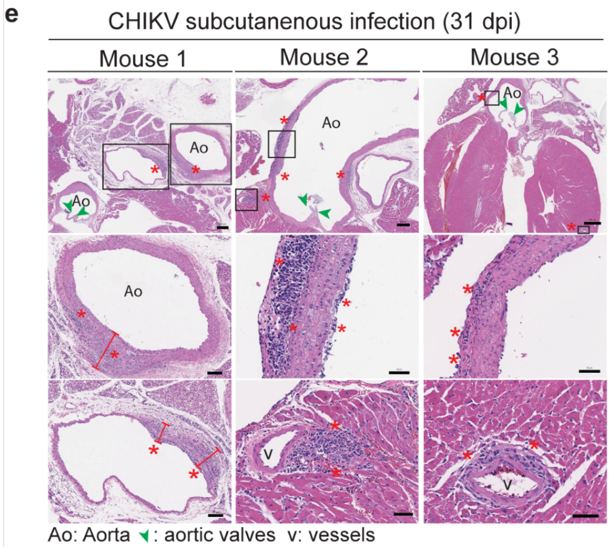

Supplementary Figure 6, related with Fig.6 and Supplementary Table 1. Representative images of more than two independent repetitions showing H&E, CD3 and, CD11b staining for intravenously inoculated CHIKV or mock-infected *Mavs*$^{-/-}$ at 15 dpi, 30 dpi, and 60 dpi, for the myocardial region **(a)** or for the large vessels attached to the base of the heart **(c)**. Inset shows the four -chamber view representation. **b and d.** Representative images of more than two independent repetitions showing H&E, CD3, and, CD11b staining for subcutaneously inoculated CHIKV or mock-infected *Mavs*$^{-/-}$ at 14 dpi, and 60 dpi, for the myocardial region **(b)** or for the large vessels attached to the base of the heart **(d)**. Inset shows the four -chamber view representation. **e.** Representatives images for three independent repetitions showing H&E staining for subcutaneously inoculated CHIKV *Mavs*$^{-/-}$ at 31 dpi. Scale bar is indicated at the bottom of each figure.

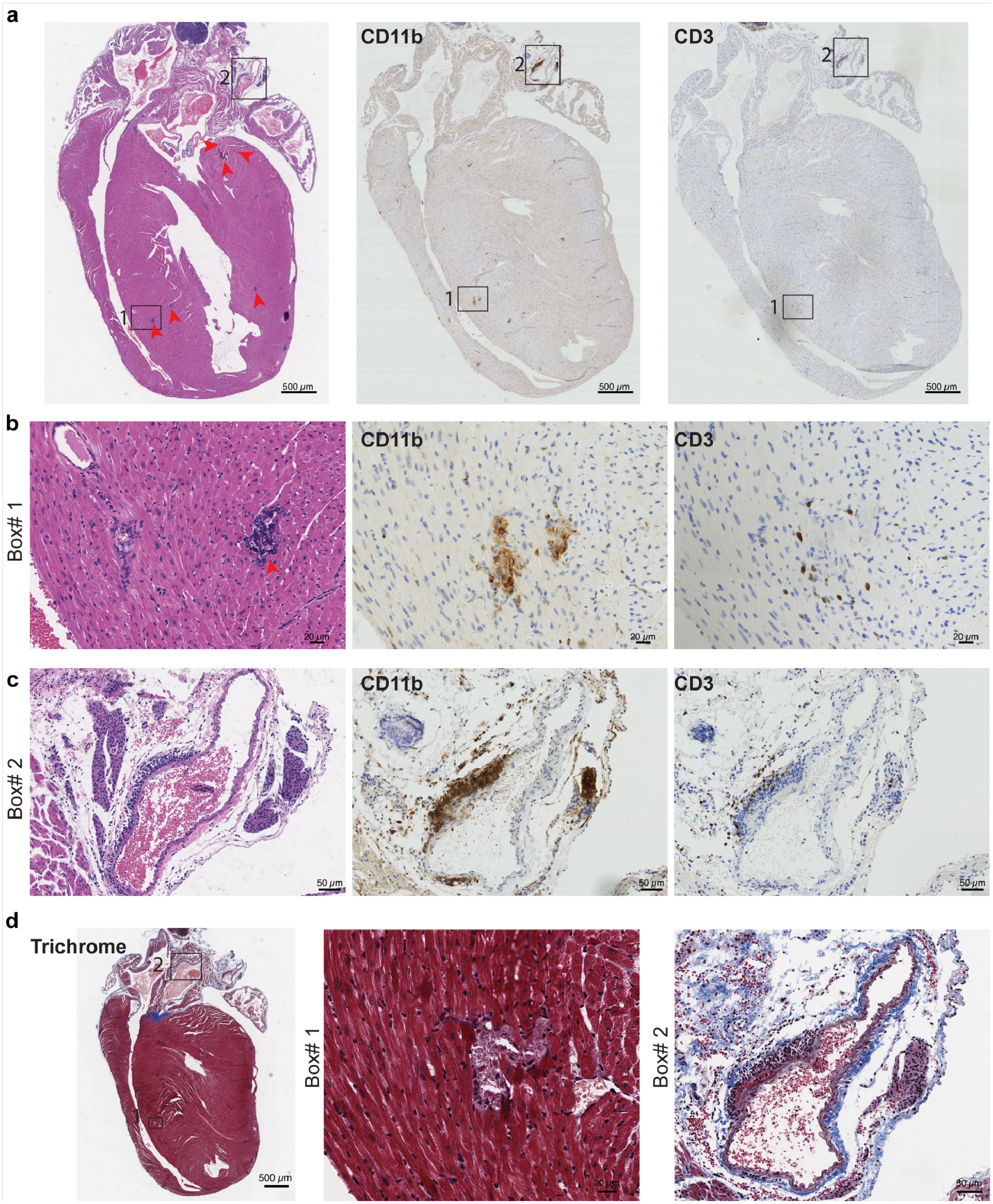

Supplementary Fig. 7, related to Supplementary Table 1. a. Four-chamber view representation of cardiac tissue from a 15 dpi *Mavs*$^{-/-}$ mouse that succumbed to CHIKV infection **(See Supplementary Table 1)**. Left panel, H&E showing four-chamber view and large vessels at the base of the heart. Middle panel: consecutive section stained with CD11b antibody. Right panel: consecutive section stained with CD3 antibody. Scale = 500 μm. **b.** Magnification of the ventricular region section (Box #1) for H&E, CD11b and CD3 staining showing infiltrates. Scale = 20 μm. **c.** Magnification of the pulmonary artery (PA) section (Box #2) for H&E, CD11b and CD3 staining. Scale = 50 μm. **d.** Mason Trichrome staining, showing no signs of fibrosis. Scale bar is indicated in each figure panel. **(a-d).** Images shown here correspond to one repetition and represent one isolated event that occurred during the study **(See Supplementary Table 1)**.

Supplementary Table 1. Disease scores of CHIKV- or mock-infected mice measured at experimental endpoint.

<table>
<tr><th>Genotype</th><th>Condition</th><th>n</th><th>Inoculation route</th><th>Dose</th><th>Experimental endpoint</th><th colspan="2">Macroscopic assessment at endpoint</th></tr>
<tr><td rowspan="2">Ifnar1-/-</td><td>CHIKV</td><td>n=11</td><td>intravenous</td><td>1E5 PFU</td><td>1 dpi</td><td rowspan="2">Unremarkable (No inflammation in the left or right hindlimb. No signs of distress)</td><td rowspan="2"><u>Notes while setting up the experimental conditions:</u> Ifnar1-/- mice infected intravenously with 1E5 PFU of CHIKV succumb to the infection between 36 to 48 hpi. Before that, they don’t show any remarkable signs of inflammation. Meanwhile, mice infected via footpad injection show extreme inflammation at the injection site noticeable after 1 dpi.</td></tr>
<tr><td>PBS-control</td><td>n=4</td><td>intravenous</td><td>1E5 PFU</td><td>1 dpi</td></tr>
<tr><td rowspan="6">Mavs-/-</td><td>CHIKV</td><td>n=20</td><td>intravenous</td><td>1E5 PFU</td><td>2 dpi/5 dpi/ 7 dpi</td><td>Unremarkable (Mild to any inflammation at the inoculation site (tail). No inflammation in the left or right hindlimb. No signs of distress.)</td><td rowspan="2"></td></tr>
<tr><td>PBS/HI-control</td><td>n=4</td><td>intravenous</td><td>1E5 PFU</td><td>2 dpi/5 dpi/7 dpi</td><td>Unremarkable</td></tr>
<tr><td>CHIKV</td><td>n=44</td><td>intravenous</td><td>1E5 PFU</td><td>10 dpi to 60 dpi</td><td>4 out of 44 animals were found dead or reached the humane endpoint. (Briefly: 2 were found dead at 10 dpi. 1 was lethargic at 14 dpi and needed to be euthanized. 1 was lethargic/immobile at 15 dpi and needed to be euthanized.). The remaining 44 animals showed no distinctive signs of disease/distress.</td><td rowspan="2"><u>Notes:</u> Histopathology of the heart was performed for one animal that succumbed to the infection (Supplementary Fig.7).</td></tr>
<tr><td>PBS/HI-control</td><td>n=12</td><td>intravenous</td><td>1E5 PFU</td><td>10 dpi to 60 dpi</td><td>Unremarkable</td></tr>
<tr><td>CHIKV</td><td>n=44</td><td>subcutaneous</td><td>1E5 PFU</td><td>10 dpi to 60 dpi</td><td>Unremarkable</td><td rowspan="2"></td></tr>
<tr><td>PBS/HI-control</td><td>n=12</td><td>subcutaneous</td><td>1E5 PFU</td><td>10 dpi to 60 dpi</td><td>Unremarkable</td></tr>
</table>

Supplementary Table 2. Pathway enrichment analysis from Hallmark dataset. For pathways enrichment analysis, genes were ranked based on -Log_{10}(FDR) values, assigning a positive or negative value depending on the direction of change. The pathway lists were obtained from the msigdbr (v7.2.1) package (Hallmark, REACTOME or KEGG) and used as input for GSEA pathway enrichment analysis through the fgsea (v1.12.0) R package. Significant regulated pathways. (FDR < 0.15 and normalized enrichment score [NES] > 0). All pathways represented in **Fig. 2f** are highlighted in bold.

	2 dpi	pval	padj	-log10(padj)	NES
1	**HALLMARK_INTERFERON_GAMMA_RESPONSE**	**0.00001**	**0.0005**	**3.30**	**1.62**
2	**HALLMARK_INTERFERON_ALPHA_RESPONSE**	**0.00002**	**0.0006**	**3.22**	**1.58**
3	HALLMARK_ALLOGRAFT_REJECTION	0.00038	0.0063	2.20	1.5
**4**	**HALLMARK_COMPLEMENT**	**0.003**	**0.032**	**1.49**	**1.44**
**5**	**HALLMARK_MTORC1_SIGNALING**	**0.003**	**0.032**	**1.49**	**1.44**
6	HALLMARK_KRAS_SIGNALING_UP	0.006	0.054	1.27	1.42
**7**	**HALLMARK_UNFOLDED_PROTEIN_RESPONSE**	**0.012**	**0.086**	**1.07**	**1.42**
**8**	**HALLMARK_INFLAMMATORY_RESPONSE**	**0.017**	**0.105**	**0.98**	**1.37**
9	HALLMARK_COAGULATION	0.019	0.108	0.97	1.39
10	HALLMARK_IL6_JAK_STAT3_SIGNALING	0.022	0.112	0.95	1.4
11	HALLMARK_EPITHELIAL_MESENCHYMAL_TRANSITION	0.033	0.127	0.90	-1.28
12	HALLMARK_MYC_TARGETS_V1	0.033	0.127	0.90	1.32
13	HALLMARK_NOTCH_SIGNALING	0.032	0.127	0.90	-1.46
14	HALLMARK_TGF_BETA_SIGNALING	0.039	0.138	0.86	-1.43
15	HALLMARK_TNFA_SIGNALING_VIA_NFKB	0.043	0.144	0.84	1.3
**26**	**HALLMARK_OXIDATIVE_PHOSPHORYLATION**	**0.211**	**0.408**	**0.39**	**-1.08**
**38**	**HALLMARK_FATTY_ACID_METABOLISM**	**0.701**	**0.922**	**0.04**	**0.89**
	5 dpi	**pval**	**padj**	**-log10(padj)**	**NES**
1	HALLMARK_ADIPOGENESIS	0.00004	0.0005	3.30	-1.72
**2**	**HALLMARK_FATTY_ACID_METABOLISM**	**0.00004**	**0.0005**	**3.30**	**-1.89**
**3**	**HALLMARK_INTERFERON_GAMMA_RESPONSE**	**0.00003**	**0.0005**	**3.30**	**1.81**
**4**	**HALLMARK_OXIDATIVE_PHOSPHORYLATION**	**0.00004**	**0.0005**	**3.30**	**-2.17**
5	HALLMARK_ALLOGRAFT_REJECTION	0.0002	0.002	2.70	1.76
6	HALLMARK_BILE_ACID_METABOLISM	0.0004	0.003	2.52	-1.64
**7**	**HALLMARK_INTERFERON_ALPHA_RESPONSE**	**0.0004**	**0.003**	**2.52**	**1.77**
8	HALLMARK_PEROXISOME	0.001	0.006	2.22	-1.6
9	HALLMARK_E2F_TARGETS	0.0033	0.017	1.77	1.66
10	HALLMARK_XENOBIOTIC_METABOLISM	0.0034	0.017	1.77	-1.45
**11**	**HALLMARK_COMPLEMENT**	**0.0099**	**0.043**	**1.37**	**1.58**
**12**	**HALLMARK_INFLAMMATORY_RESPONSE**	**0.0102**	**0.043**	**1.37**	**1.57**
13	HALLMARK_G2M_CHECKPOINT	0.0129	0.049	1.31	1.53
**14**	**HALLMARK_MTORC1_SIGNALING**	**0.0158**	**0.053**	**1.28**	**1.5**
15	HALLMARK_TGF_BETA_SIGNALING	0.0149	0.053	1.28	-1.49
16	HALLMARK_PI3K_AKT_MTOR_SIGNALING	0.0177	0.055	1.26	1.5
17	HALLMARK_IL6_JAK_STAT3_SIGNALING	0.0303	0.089	1.05	1.46
18	HALLMARK_MITOTIC_SPINDLE	0.0416	0.104	0.98	1.37
19	HALLMARK_TNFA_SIGNALING_VIA_NFKB	0.0377	0.104	0.98	1.38
**20**	**HALLMARK_UNFOLDED_PROTEIN_RESPONSE**	**0.0417**	**0.104**	**0.98**	**1.4**

21	HALLMARK_GLYCOLYSIS	0.0469	0.112	0.95	-1.26
22	HALLMARK_MYOGENESIS	0.0509	0.116	0.94	-1.25
23	HALLMARK_IL2_STAT5_SIGNALING	0.0585	0.127	0.90	1.33
24	HALLMARK_ANDROGEN_RESPONSE	0.063	0.131	0.88	1.36

Supplementary Table 3. Pathway enrichment analysis from Reactome dataset. For pathways enrichment analysis, genes were ranked based on -Log_{10}(FDR) values, assigning a positive or negative value depending on the direction of change. The pathway lists were obtained from the msigdbr (v7.2.1) package (Hallmark, REACTOME or KEGG) and used as input for GSEA pathway enrichment analysis through the fgsea (v1.12.0) R package. Significant regulated pathways. (FDR < 0.15 and normalized enrichment score [NES] > 0).

	2 dpi	pval	padj	-log10(padj)	NES
**1**	**REACTOME_ADAPTIVE_IMMUNE_SYSTEM**	**0.00001**	**0.0059**	2.23	**1.43**
**2**	**REACTOME_INNATE_IMMUNE_SYSTEM**	**0.00001**	**0.0059**	2.23	**1.47**
**3**	**REACTOME_CYTOKINE_SIGNALING_IN_IMMUNE_SYSTEM**	**0.00003**	**0.009**	2.05	**1.37**
**4**	**REACTOME_NEUTROPHIL_DEGRANULATION**	**0.00003**	**0.009**	2.05	**1.47**
5	REACTOME_IMMUNOREGULATORY_INTERACTIONS_ BETWEEN_A_LYMPHOID_AND_A_NON_LYMPHOID_CELL	0.00004	0.01	2.00	1.57
**6**	**REACTOME_INTERFERON_SIGNALING**	**0.00006**	**0.01**	2.00	**1.55**
7	REACTOME_CELLULAR_RESPONSES_TO_EXTERNAL_ STIMULI	0.0004	0.05	1.30	1.36
8	REACTOME_COLLAGEN_BIOSYNTHESIS_AND_MODIFYING_ENZYMES	0.0003	0.05	1.30	-1.73
9	REACTOME_DEUBIQUITINATION	0.0003	0.05	1.30	1.45
10	REACTOME_COMPLEMENT_CASCADE	0.001	0.1	1.00	1.54
11	REACTOME_SIGNALING_BY_INTERLEUKINS	0.0009	0.1	1.00	1.38
12	REACTOME_INFECTIOUS_DISEASE	0.0013	0.11	0.96	1.3
13	REACTOME_METABOLISM_OF_RNA	0.0011	0.11	0.96	1.31
14	REACTOME_SIGNALING_BY_CYTOSOLIC_FGFR1_FUSION_MUTANTS	0.0013	0.11	0.96	1.46
15	REACTOME_UB_SPECIFIC_PROCESSING_PROTEASES	0.0012	0.11	0.96	1.46
16	REACTOME_CLASS_I_MHC_MEDIATED_ANTIGEN_PROCESSING_PRESENTATION	0.0018	0.13	0.89	1.38
17	REACTOME_HIV_INFECTION	0.0021	0.14	0.85	1.44
18	REACTOME_INTERFERON_ALPHA_BETA_SIGNALING	0.0023	0.14	0.85	1.51
19	REACTOME_MISCELLANEOUS_TRANSPORT_AND_BINDING_EVENTS	0.0023	0.14	0.85	-1.6
	5 dpi	pval	padj	-log10(padj)	NES
1	REACTOME_ADAPTIVE_IMMUNE_SYSTEM	**2.17E-05**	**0.00622031**	**2.21**	**1.62**
**2**	**REACTOME_MITOCHONDRIAL_PROTEIN_IMPORT**	**2.67E-05**	**0.00622031**	**2.21**	**-1.77**
3	REACTOME_PROTEIN_LOCALIZATION	3.67E-05	0.00622031	2.21	-1.83
**4**	**REACTOME_PYRUVATE_METABOLISM_AND_CITRIC_ACID_TCA_CYCLE**	**2.56E-05**	**0.00622031**	**2.21**	**-1.82**
**5**	**REACTOME_RESPIRATORY_ELECTRON_TRANSPORT**	**2.91E-05**	**0.00622031**	**2.21**	**-1.91**
6	REACTOME_RESPIRATORY_ELECTRON_TRANSPORT_ATP_SYNTHESIS_BY_CHEMIOSM OTIC_COUPLING_AND_HEAT_PRODUCTION_BY_UNCOUPLING_PROTEINS_	3.14E-05	0.00622031	2.21	-1.98
7	REACTOME_THE_CITRIC_ACID_TCA_CYCLE_AND_RESPIRATORY_ELECTRON_ TRANSPORT	3.62E-05	0.00622031	2.21	-2.1
8	REACTOME_COMPLEX_I_BIOGENESIS	5.05E-05	0.00666532	2.18	-1.75
9	REACTOME_TRANSLATION	5.06E-05	0.00666532	2.18	-1.66
**10**	**REACTOME_METABOLISM_OF_AMINO_ACIDS_AND_DERIVATIVES**	**6.05E-05**	**0.00717182**	**2.14**	**-1.59**
11	REACTOME_IMMUNOREGULATORY_INTERACTIONS_BETWEEN_A_LYMPHOID_AND_A_N ON_LYMPHOID_CELL	0.00013048	0.01405648	1.85	1.78
**12**	**REACTOME_INNATE_IMMUNE_SYSTEM**	**0.00022103**	**0.0201481**	**1.70**	**1.49**
**13**	**REACTOME_MITOCHONDRIAL_FATTY_ACID_BETA_OXIDATION**	**0.00021913**	**0.0201481**	**1.70**	**-1.69**
14	REACTOME_PEROXISOMAL_PROTEIN_IMPORT	0.00024398	0.02065076	1.69	-1.71
15	REACTOME_SIGNALING_BY_RETINOIC_ACID	0.00056378	0.04453868	1.35	-1.65
16	REACTOME_MITOCHONDRIAL_BIOGENESIS	0.00062986	0.04664865	1.33	-1.65
17	REACTOME_KERATINIZATION	0.00073634	0.05132701	1.29	-1.65

18	REACTOME_CRISTAE_FORMATION	0.00095672	0.05966877	1.22	-1.63
19	REACTOME_FORMATION_OF_THE_CORNIFIED_ENVELOPE	0.00094477	0.05966877	1.22	-1.65
20	REACTOME_GLYOXYLATE_METABOLISM_AND_GLYCINE_DEGRADATION	0.00115026	0.06815301	1.17	-1.62
21	REACTOME_CLASS_C_3_METABOTROPIC_GLUTAMATE_PHEROMONE_RECEPTORS_	0.00135993	0.07673876	1.11	1.49
22	REACTOME_MOLECULES_ASSOCIATED_WITH_ELASTIC_FIBRES	0.00167998	0.09048965	1.04	-1.61
23	REACTOME_CITRIC_ACID_CYCLE_TCA_CYCLE_	0.00191307	0.09445767	1.02	-1.57
24	REACTOME_PYRUVATE_METABOLISM	0.00187531	0.09445767	1.02	-1.59
**25**	**REACTOME_CYTOKINE_SIGNALING_IN_IMMUNE_SYSTEM**	**0.00243127**	**0.115242**	**0.94**	**1.44**
26	REACTOME_PHASE_II_CONJUGATION_OF_COMPOUNDS	0.00304632	0.13526861	0.87	-1.56
27	REACTOME_STING_MEDIATED_INDUCTION_OF_HOST_IMMUNE_RESPONSES	0.00308207	0.13526861	0.87	1.49

Supplementary Table 4. Pathway enrichment analysis from KEGG dataset. For pathways enrichment analysis, genes were ranked based on -Log$_{10}$(FDR) values, assigning a positive or negative value depending on the direction of change. The pathway lists were obtained from the msigdbr (v7.2.1) package (Hallmark, REACTOME or KEGG) and used as input for GSEA pathway enrichment analysis through the fgsea (v1.12.0) R package. Significant regulated pathways. (FDR < 0.15 and normalized enrichment score [NES] > 0). Pathways represented in **Supplementary Fig. 3c** are highlighted in bold.

	2 dpi	pval	padj	-log10(padj)	NES
1	KEGG_SYSTEMIC_LUPUS_ERYTHEMATOSUS	0.0004	0.07	1.15	1.55
2	KEGG_COMPLEMENT_AND_COAGULATION_CASCADES	0.0015	0.14	0.85	1.53
	5 dpi	pval	padj	-log10(padj)	NES
1	KEGG_ALZHEIMERS_DISEASE	0.000036	0.002	2.70	-1.92
2	KEGG_HUNTINGTONS_DISEASE	0.000038	0.002	2.70	-1.94
3	KEGG_OXIDATIVE_PHOSPHORYLATION	0.000031	0.002	2.70	-1.96
4	KEGG_PARKINSONS_DISEASE	0.000031	0.002	2.70	-1.82
5	KEGG_VALINE_LEUCINE_AND_ISOLEUCINE_DEGRADATION	0.000074	0.003	2.52	-1.73
6	KEGG_CITRATE_CYCLE_TCA_CYCLE	0.0001	0.004	2.40	-1.68
7	KEGG_FATTY_ACID_METABOLISM	0.0002	0.005	2.30	-1.69
8	KEGG_BUTANOATE_METABOLISM	0.0003	0.007	2.15	-1.65
**9**	**KEGG_CARDIAC_MUSCLE_CONTRACTION**	**0.0009**	**0.015**	**1.82**	**-1.64**
10	KEGG_PEROXISOME	0.0008	0.015	1.82	-1.64
11	KEGG_PROPANOATE_METABOLISM	0.0007	0.015	1.82	-1.63
12	KEGG_GLYCOLYSIS_GLUCONEOGENESIS	0.0018	0.027	1.57	-1.62
13	KEGG_PYRUVATE_METABOLISM	0.0025	0.036	1.44	-1.6
14	KEGG_SULFUR_METABOLISM	0.0039	0.052	1.28	-1.49
15	KEGG_ALLOGRAFT_REJECTION	0.0082	0.073	1.14	1.53
16	KEGG_AMINOACYL_TRNA_BIOSYNTHESIS	0.0073	0.073	1.14	-1.55
17	KEGG_AUTOIMMUNE_THYROID_DISEASE	0.0079	0.073	1.14	1.54
18	KEGG_CELL_ADHESION_MOLECULES_CAMS	0.0083	0.073	1.14	1.61
19	KEGG_CYTOSOLIC_DNA_SENSING_PATHWAY	0.0068	0.073	1.14	1.58
20	KEGG_NATURAL_KILLER_CELL_MEDIATED_CYTOTOXICITY	0.0072	0.073	1.14	1.63
21	KEGG_NEUROACTIVE_LIGAND_RECEPTOR_INTERACTION	0.0082	0.073	1.14	1.6
22	KEGG_GRAFT_VERSUS_HOST_DISEASE	0.0101	0.081	1.09	1.53
23	KEGG_T_CELL_RECEPTOR_SIGNALING_PATHWAY	0.01	0.081	1.09	1.58
**24**	**KEGG_ARRHYTHMOGENIC_RIGHT_VENTRICULAR_CARDIOMYOPATHY_ARVC**	**0.0134**	**0.083**	**1.08**	**-1.47**
25	KEGG_B_CELL_RECEPTOR_SIGNALING_PATHWAY	0.0121	0.083	1.08	1.55
26	KEGG_CHEMOKINE_SIGNALING_PATHWAY	0.0135	0.083	1.08	1.54
27	KEGG_PPAR_SIGNALING_PATHWAY	0.0109	0.083	1.08	-1.49
28	KEGG_RIBOSOME	0.0132	0.083	1.08	-1.46
29	KEGG_SYSTEMIC_LUPUS_ERYTHEMATOSUS	0.012	0.083	1.08	1.55

**30**	**KEGG_VIRAL_MYOCARDITIS**	**0.0133**	**0.083**	**1.08**	**1.54**
31	KEGG_FC_GAMMA_R_MEDIATED_PHAGOCYTOSIS	0.0143	0.085	1.07	1.53
32	KEGG_VALINE_LEUCINE_AND_ISOLEUCINE_BIOSYNTHESIS	0.0158	0.091	1.04	-1.42
33	KEGG_BETA_ALANINE_METABOLISM	0.0182	0.102	0.99	-1.48
34	KEGG_BASAL_CELL_CARCINOMA	0.0199	0.105	0.98	-1.46
35	KEGG_RNA_POLYMERASE	0.02	0.105	0.98	-1.48
36	KEGG_LYSINE_DEGRADATION	0.0246	0.126	0.90	-1.46
37	KEGG_TOLL_LIKE_RECEPTOR_SIGNALING_PATHWAY	0.026	0.129	0.89	1.47
38	KEGG_PRIMARY_IMMUNODEFICIENCY	0.028	0.132	0.88	1.46
39	KEGG_RIG_I_LIKE_RECEPTOR_SIGNALING_PATHWAY	0.0279	0.132	0.88	1.47
40	KEGG_JAK_STAT_SIGNALING_PATHWAY	0.0292	0.134	0.87	1.44
41	KEGG_CELL_CYCLE	0.0308	0.137	0.86	1.43
42	KEGG_TRYPTOPHAN_METABOLISM	0.0312	0.137	0.86	-1.44
43	KEGG_CALCIUM_SIGNALING_PATHWAY	0.0324	0.139	0.86	-1.31
