# Supplementary material for: MAVS signaling is required for preventing persistent chikungunya heart infection and chronic vascular tissue inflammation
Source: Nat Commun. 2023 Aug 3;14:4668. doi: 10.1038/s41467-023-40047-w (PMC10400619; doi:10.1038/s41467-023-40047-w)
Supplement: Supplementary file 3 — Description of Additional Supplementary files [file 41467_2023_40047_MOESM3_ESM.pdf]

<u>Description of Additional Supplementary Files</u>

File name: Supplementary Data 1
Description: This dataset contains the histopathological analysis of H&E heart sections for WT, *Mavs*$^{+/-}$ and *Mavs*$^{-/-}$ CHIKV-infected and mock-infected mice.
Microscopy image raw data is stored in OMERO Plus and is accessible through the NYU Data Catalog: https://datacatalog.med.nyu.edu/dataset/10621.

File name: Supplementary Data 2
Description: List of differentially expressed genes at 2 dpi. Differential RNA expression analysis was performed using DEseq2. The Log_2FC represents the log_2 of the fold change between normalized counts of each group. P values were calculated using the Wald test, between CHIKV-infected and heat inactivated (HI) control groups, followed by multiple hypothesis correction via the Benjamini-Hochberg method for calculating the false discovery rate (FDR) values. Significant expression changes were defined as FDR < 0.15 and absolute Log2FC > 1.

File name: Supplementary Data 3
Description: List of differentially expressed genes at 5 dpi. Differential RNA expression analysis was performed using DEseq2. The Log_2FC represents the log_2 of the fold change between normalized counts of each group. P values were calculated using the Wald test, between CHIKV-infected and heat inactivated (HI) control groups, followed by multiple hypothesis correction via the Benjamini-Hochberg method for calculating the false discovery rate (FDR) values. Significant expression changes were defined as FDR < 0.15 and absolute Log2FC > 1.
